# Do Brazilian medical journals reference Brazilian articles? A cross-sectional study

**DOI:** 10.1590/1677-5449.202200011

**Published:** 2022-06-03

**Authors:** Daniela Ferreira Tramontin, Luís Vinícius Pires da Costa, Antônio Leonardo Jahati Cavalcante Pimentel, Rafael Silva Lemos, Maria Eduarda dos Santos Lopes Vasconcelos, Lívia Guerreiro de Barros Bentes, Nayara Pontes de Araújo, Rui Sérgio Monteiro de Barros

**Affiliations:** 1 Universidade do Estado do Pará – UEPA, Belém, PA, Brasil.; 2 Centro Universitário do Pará – CESUPA, Belém, PA, Brasil.; 3 Universidade Federal do Pará – UFPA, Belém, PA, Brasil.; 4 Universidade Federal do Rio de Janeiro – UFRJ, Rio de Janeiro, RJ, Brasil.

**Keywords:** bibliography, journal article, impact factor, journal impact factor

## Abstract

**Background:**

The quantity and quality of Brazilian scientific output increases decade by decade. However, there is a tendency to undervalue Brazilian journals, illustrated by the low number of citations compared with texts in international journals, with the tacit justification that foreign articles are of superior quality.

**Objectives:**

To investigate the differences in numbers of citations of Brazilian and international periodicals in three Brazilian journals from 2016 to 2020.

**Methods:**

All articles published in the Journal of the Brazilian College of Surgeons, in the Jornal Vascular Brasileiro, and in Acta Cirúrgica Brasileira from 2016 to 2020 were analyzed. The references of these studies were analyzed, summing the total number of citations and classifying them as published in Brazilian or foreign journals.

**Results:**

A total of 902 articles were analyzed, totaling 23,394 references, with a mean of 25.81 ± 8.59 references per article. Of these, 2,680 (11.45%) were Brazilian, equating to a mean of 2.95 ± 3.79 Brazilian references per article.

**Conclusions:**

It is necessary to improve appreciation of Brazilian periodicals, especially among Brazilian researchers and institutions responsible for science funding.

## INTRODUCTION

Scientific communication can be defined as a group of activities related to production, dissemination, and use of information.^1^ This information is based on knowledge, which, when described in a formal manner, is primarily disseminated via scientific journals.^2^^,^ 3 The spread of internet access provided an unparalleled strategy for raising the visibility of science with on-line versions of periodicals, thus bringing both the specialist and non-specialist audiences into contact with scientific knowledge and enabling creation and consolidation of digital information databases, an important means of propagating the fruit of scientific study.^4^

The quantity and quality of Brazilian scientific output increases decade by decade.^5^ A significant part of this growth can be attributed to increased publication in journals indexed on information databases; part is due to increased citation of Brazilian articles.^6^^,^ 7 This expansion is also linked to the exponential growth of Masters and Doctoral degree programs, combined with the increased collaboration between researchers and the historic support from the research funding agency Coordenação de Aperfeiçoamento de Pessoal de Nível Superior (CAPES), which plays a decisive role in supporting scientific innovations in Brazil.^8^

One of the parameters that CAPES uses to evaluate postgraduate programs is the Qualis ratings of the periodicals in which their articles are published: a list classifying the quality of the journals in which postgraduate production is published.^9^ This classification does not illustrate the scientific repercussions of studies as clearly as impact factors (IF),^10^ but does nevertheless have considerable influence on which periodicals are chosen for publication, since publishing in journals with higher Qualis classifications can ensure that researchers will win or maintain funding.^11^

Evidence that Brazilian journals are awarded lower Qualis ratings than international periodicals supports criticisms that CAPES gives preference to where an article is published rather than to the quality of its content, resulting in a disincentive and devaluation of publication in Brazilian journals.^10^^,^ 12 In such a scenario, the undervaluing of Brazilian journals is manifest in the low numbers of citations of articles published in Brazilian journals compared with texts in international ones, with the tacit justification that foreign articles are of superior quality.^13^

A review of the literature did not identify any recent studies that quantify the imbalance between citation of international and Brazilian articles in Brazilian journals, in favor of periodicals from other countries. The objective of this study was therefore to determine the differences between the numbers of citations of Brazilian and international journals in articles published in three Brazilian journals from 2016 to 2020.

## METHODOLOGY

This cross-sectional, observational study analyzed references in three Brazilian surgery journals: the Journal of the Brazilian College of Surgeons (JBC), the Jornal Vascular Brasileiro (JVB), and Acta Cirúrgica Brasileira (ACB).

All articles published in these journals from 2016 to 2020 were analyzed. The inclusion criteria were all references cited in original articles. Exclusion criteria were studies classified as editorials, case reports, technical notes, therapeutic challenges, errata, and literature reviews.

The references of the selected studies were then evaluated, summing the total number of citations, the numbers of references to Brazilian journals and foreign journals, and the ratio between them. References to books, internet sites, dissertations, and citations of citations (apud) were ignored.

Statistical analysis was conducted with the Kruskal-Wallis test of differences between groups, with values of p < 0.05 considered significant. Since these journals are all in the public domain, there was no need to submit the project to a Research Ethics Committee.

## RESULTS

After data collection, 902 articles from the three periodicals selected were analyzed, totaling 23,394 references, with a mean of 25.81 ± 8.59 per article. Of these, 2,680 (11.45%) were Brazilian, equating to a mean of 2.95 ± 3.79 Brazilian references per article.

Analyzing the references by year, the largest numbers of studies (204) and citations (5,087) were in 2016; followed by 2018, with 196 articles and 4,995 references, 2017, with 180 articles and 4,610 references; 2019, with 174 studies and 4,477 citations; and 2020, with 148 studies and 4,225 citations. With regard to the proportions of Brazilian and foreign citations, 2016 was the year with the highest proportion of Brazilian citations, at 12.67% of the total, while the proportion was lowest in 2020 (10.88%).

**Figure 1:**
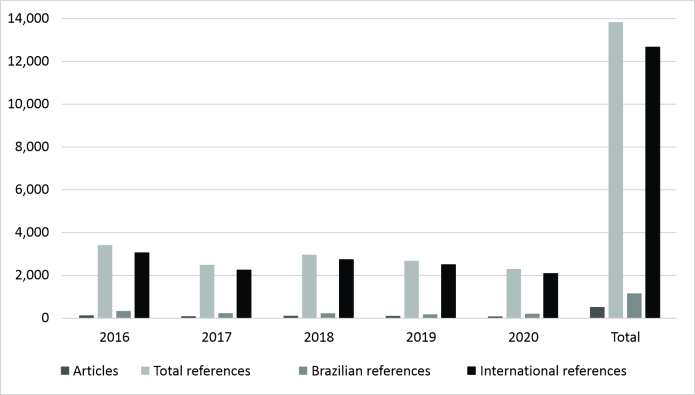
Total numbers of articles and references in Acta Cirúrgica Brasileira (2016-2020). Source: study protocol.

With regard to results for the three different journals, as shown in Tables 1 and 2 and Figure 1, ACB had the greatest number of articles and citations during the period analyzed, at 507 (56.20%) articles and 13,835 (59.13%) references included according to the research protocol. Additionally, the same journal cited the lowest proportion of Brazilian studies (8.40%), when compared to the others, and 438 (86.39%) of the articles in ACB cited from 0 to 20% of their references from Brazilian journals, with a statistically significant difference (p < 0.05).

**Table 1:** Numbers of articles, total references, and Brazilian references in the journals analyzed (2016-2020).

**Year**	**Brazilian References**						**Total references**			**Total articles**		
	**ACB**		**JVB**		**JBC**		**ACB**	**JVB**	**JBC**	**ACB**	**JVB**	**JBC**
	**N**	**%**	**N**	**%**	**N**	**%**	**N**	**N**	**N**	**N**	**N**	**N**
2016	333	9.77	108	23.2	204	16.76	3,405	465	1,217	130	22	52
2017	226	9.10	77	17.03	243	14.49	2,481	452	1,677	93	21	66
2018	219	7.38	56	16.32	250	14.81	2,965	343	1,687	111	17	68
2019	171	6.38	111	23.17	222	16.84	2,680	479	1,318	98	19	57
2020	214	9.28	93	15.81	153	11.47	2,304	588	1,333	75	24	49
Total	1,163	8.40	445	19.12	1,072	14.82	13,835	2,327	7,232	507	103	292

Source: study protocol.

ACB = Acta Cirúrgica Brasileira; JVB = Journal Vascular Brasileiro; JBC = Journal of the Brazilian College of Surgeons; N = number.

**Table 2:** Brazilian references as a proportion of the total in the journals analyzed (2016-2020).

**Brazilian references as a proportion of the total (%)**	**ACB**		**JBV**		**JBC**	
	**N**	**%**	**N**	**%**	**N**	**%**
0.00	200	39.45	24	23.30	54	18.94
1.00-20.00	238	46.94	40	38.83	156	53.42
21.00-40.00	53	10.45	25	24.27	63	21.58
41.00-60.00	11	2.17	9	8.74	15	5.14
61.00-80.00	1	0.20	3	2.91	3	1.03
81.00-100.00	4	0.79	2	1.94	1	0.34
**Total**	**507**	**100.00**	**103**	**100.00**	**292**	**100.00**

Source: study protocol.

ACB = Acta Cirúrgica Brasileira; JVB = Journal Vascular Brasileiro; JBC = Journal of the Brazilian College of Surgeons; N = number.

**Figure 2:**
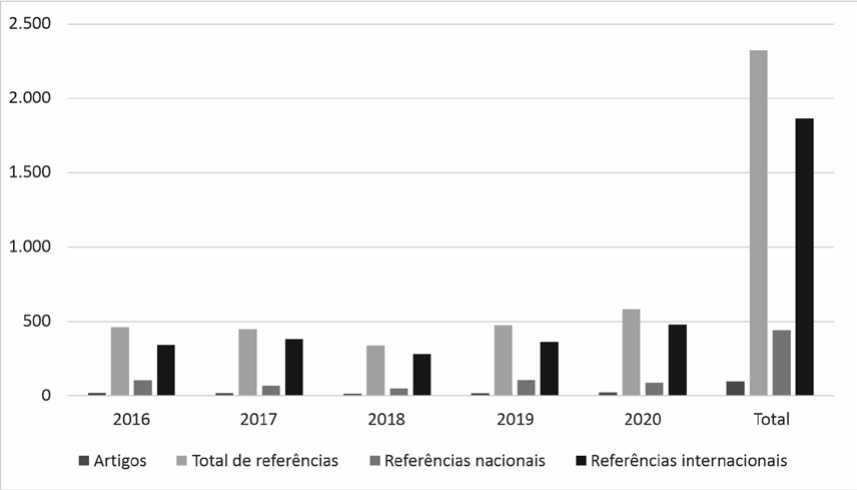
Total numbers of articles and references in the Jornal Vascular Brasileiro (2016-2020). Source: study protocol.

The JVB accounted for 103 (11.41%) articles and 2,327 (9.94%) of the references, as illustrated in Figure 2. This journal had a higher proportion of citations of Brazilian studies (19.12%) than the other two, with 64 (62.13%) articles in which 0 to 20% of the references were to articles in Brazilian journals.

**Figure 3:**
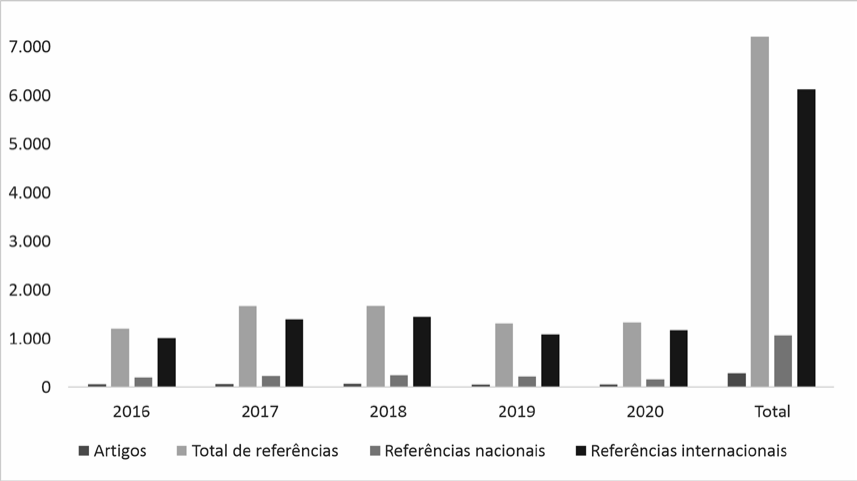
Total numbers of articles and references in the Journal of the Brazilian College of Surgeons (2016-2020). Source: study protocol.

The JBC had 292 (32.37%) studies and 7,232 (30.91%) citations (Figure 3). The proportion of Brazilian references in this journal was 11.47%, and 210 (71.92%) articles had 0 to 20% of references to articles from Brazilian journals.

It is also notable that 30.59% of the articles analyzed did not have any Brazilian references whatsoever. Broken down by journal, 23.30% of the articles in the JVB and 17.80% of the articles in the JBC had no Brazilian studies, while Acta Cirúrgica Brasileira was the journal with the highest proportion of articles with entirely foreign references (39.44%).

## DISCUSSION

The process of Brazilian scientific output is recent and is still in consolidation. In addition to the late construction of University education, the country’s educational tradition is still deficient and outdated, prioritizing techniques based on presentation and memorization over a constructive and critical education.^14^ However, international indicators demonstrate that Brazil has made considerable advances in scientific fields over the last four decades: at the start of the 2000s, Brazil was rated as having the sixth best performance out of the 30 countries with greatest prominence in the Institute for Scientific Information (ISI) world science rankings, behind South Korea, Taiwan, China, Spain, and Turkey.^15^

In medicine, however, Brazilian scientific publications tend to give greater distinction to international publications, especially those in English and from the United States, in detriment to Brazilian work. Despite the relevance of their publication, Brazilian journals are read little, with low IFs on the world stage.^16^^,^ 17

This study confirms that this tendency has been maintained over recent years. Indeed, in the journals analyzed, there was even a drop in the proportion of Brazilian citations, from 12.67% in 2016 to 10.88% in 2020. International publications even account for a large majority (80.88%) of citations in the journal with the highest proportion of Brazilian references (the Jornal Vascular Brasileiro).

On this subject, Petroianu^18^ points out that there are many high quality studies published in Brazilian journals that are unable even to attract the attention of Brazilian researchers, which he classifies as a colonialist and self-destructive tendency. Extending this analysis, Petroianu also states that even those Brazilian authors supported by Brazilian public funding end up benefiting other countries when they prefer their journals, with no benefit for local science. It should be understood that this pattern is reinforced by the agencies responsible for assessing Brazilian scientific institutions, since they prioritize the IF of the journals in which institutions’ studies are published, thereby discouraging publication in smaller Brazilian journals.

With regard to the consequences of this disdain, Meneghini^19^ argues that although English is the dominant language, using it can make it difficult for readers and users, such as health professionals and journalists, to access biomedical publications, especially in developing countries. Difficulty understanding international publications is of particular concern in studies with clinical implications, since it may deprive these professionals’ patients of the benefits. Furthermore, the discrepancy between national and international scientific productions should also be considered, particularly with respect to specific areas of the literature, since it disproportionately prioritizes citation of international articles, because of the vast production in comparison with Brazilian literature.

Another problem is the tendency to ignore extremely relevant publications because of the language of publication. For example, German scientists had already identified a significant causal relationship between smoking and lung cancer at the start of the 1930s, which was ignored by the scientific community for more than three decades, until British and American scientists rediscovered the link and stimulated public policies against cigarettes.^20^

In a study similar to this one, Teixeira et al.^21^ point out that although the practice of citing international periodicals cannot be considered wrong, it creates a perpetual cycle of disdain for Brazilian research. Teixeira et al. also argue that the IF of the Brazilian journals analyzed increased from 2007 to 2011, which illustrates the high quality of Brazilian research. Moreover, the lack of, or recent, indexing of Brazilian journals on search databases such as PubMed, developed by the National Center for Biotechnology Information (NCBI), and others, worsens the problem of the invisibility of Brazilian scientific output, preventing this literature from being accessed by Brazilian and international researchers and further reducing the impact of Brazilian scientific production.

The existence of a ‘lingua franca’ is not a novelty, since they have often existed in the history of humanity, generally for commercial and diplomatic reasons. French was the language of diplomacy in seventeenth century Europe and is still used in international institutions today. Thus, since English facilitates communication between researchers, learning it should be encouraged in the scientific community.^22^

However, encouraging understanding of international periodicals does not imply ignoring the need to value domestic scientific language. Publication of bilingual and multilingual articles is a potential solution that is inexpensive and has been offered for years by the Scientific Electronic Library On-line (SciELO), an electronic library founded in 1997 by the Fundação de Amparo à Pesquisa do Estado de São Paulo (FAPESP – the São Paulo state research support agency) in partnership with the Latin American and Caribbean Center on Health Sciences Information (BIREME).^23^

Ideally, both Brazilian and international journals should consider publishing at least two versions of articles – one in English and another in the authors’ native language. Brazilian agencies, publishers, and authors need to work in conjunction to expand bilingual and multilingual publications and raise the visibility of Brazilian research. Notwithstanding, Brazilian authors themselves could and should make efforts to cite Brazilian studies, when relevant, and it falls to the editorial teams of Brazilian journals to correct omissions related to citation of local studies.

## CONCLUSIONS

There has been an evident increase in the quality of Brazilian research over recent years, but there is nonetheless a significant difference compared to the number of citations of international periodicals, in detriment to Brazilian periodicals. From this perspective, there is a need to value Brazilian journals more, especially on the part of Brazilian researchers and the institutions responsible for fostering science, in order to continually incentivize knowledge production.
