# Supplementary material for: Do Brazilian medical journals reference Brazilian articles? A cross-sectional study
Source: J Vasc Bras. 2022 Jun 3;21:e20220001. doi: 10.1590/1677-5449.202200011 (PMC9222147; doi:10.1590/1677-5449.202200011)
Supplement: Translation

# Translation: Os periódicos médicos brasileiros referenciam artigos nacionais? Um estudo transversal

**DOI:** 10.1590/1677-5449.202200012

**Authors:** Daniela Ferreira Tramontin, Luís Vinícius Pires da Costa, Antônio Leonardo Jahati Cavalcante Pimentel, Rafael Silva Lemos, Maria Eduarda dos Santos Lopes Vasconcelos, Lívia Guerreiro de Barros Bentes, Nayara Pontes de Araújo, Rui Sérgio Monteiro de Barros

**Affiliations:** 1 Universidade do Estado do Pará – UEPA, Belém, PA, Brasil.; 2 Centro Universitário do Pará – CESUPA, Belém, PA, Brasil.; 3 Universidade Federal do Pará – UFPA, Belém, PA, Brasil.; 4 Universidade Federal do Rio de Janeiro – UFRJ, Rio de Janeiro, RJ, Brasil.

**Keywords:** bibliografia, artigo de revista, fator de impacto, fator de impacto de revistas

## Abstract

**Contexto:**

A produção científica brasileira cresce de forma quantitativa e qualitativa a cada década. Entretanto, há uma desvalorização dos periódicos brasileiros demonstrada através da baixa quantidade de citações em comparação a textos de revistas internacionais, com justificativa subentendida de que há superioridade qualitativa de artigos estrangeiros.

**Objetivos:**

Verificar a diferença entre o número de citações de periódicos nacionais e internacionais em três revistas brasileiras no período de 2016 a 2020.

**Métodos:**

Foram analisados todos os artigos publicados na Revista do Colégio Brasileiro de Cirurgiões, no Jornal Vascular Brasileiro e na Acta Cirúrgica Brasileira no intervalo de 2016 a 2020. Os estudos selecionados tiveram suas referências avaliadas, sendo contabilizado o número total de citações e se eram provenientes de periódicos brasileiros ou de periódicos estrangeiros.

**Resultados:**

Foram analisados 902 artigos, totalizando 23.394 referências, com média de 25,81 ± 8,59 por artigo. Nesse contexto, 2.680 (11,45%) eram nacionais, correspondentes a uma média de 2,95 ± 3,79 referências brasileiras por artigo.

**Conclusões:**

Faz-se necessária a maior valorização dos periódicos brasileiros, em especial por parte dos pesquisadores nacionais e das instituições responsáveis pelo fomento da ciência.

## INTRODUÇÃO

A comunicação científica pode ser definida como o agrupamento de atividades relacionadas à produção, à disseminação e ao uso da informação^1^. Essas informações são baseadas em conhecimentos que, quando descritos de maneira formal, têm o periódico científico como principal canal de disseminação^2^^,^ 3. A difusão do acesso à internet constituiu estratégia ímpar para maior visibilidade da ciência com a versão on-line dos periódicos, aproximando tanto o público especializado quanto o não especializado do conhecimento científico, assim como permitindo a criação e a solidificação das bases de dados informacionais digitais, importante meio de propagação do estudo científico^4^.

A produção científica brasileira cresce de forma quantitativa e qualitativa a cada década^5^. Uma parte importante desse crescimento é atribuída ao aumento da publicação em periódicos indexados em bases de dados informacionais; outra parte se dá devido ao aumento da citação de artigos brasileiros^6^^,^ 7. Ainda, essa expansão se relaciona à evolução exponencial dos programas de pós-graduação *stricto sensu* associada ao aumento da colaboração entre pesquisadores e ao apoio histórico advindo da Coordenação de Aperfeiçoamento de Pessoal de Nível Superior (CAPES), órgão determinante no impulso das inovações científicas no Brasil^8^.

Entre os parâmetros utilizados pela CAPES para avaliação dos programas de pós-graduação *stricto sensu*, está o Qualis dos periódicos em que os artigos são publicados: uma lista de classificação da qualidade das revistas em que as produções de pós-graduação serão publicadas^9^. Essa classificação traduz menos a realidade da repercussão científica dos estudos quando comparada ao fator de impacto (FI)^10^, mas, ainda assim, possui grande influência no momento da escolha dos periódicos em que serão feitas as publicações, tendo em vista que uma maior classificação Qualis da revista na qual o artigo será publicado pode garantir ao pesquisador o ganho ou a manutenção de determinado recurso financeiro^11^.

As evidências de que periódicos nacionais possuem valor de Qualis menor do que periódicos internacionais materializam as críticas recebidas pela CAPES devido à preferência na avaliação do local onde o artigo é publicado em detrimento da qualidade do seu conteúdo, implicando em desincentivo e desvalorização da publicação em periódicos nacionais^10^^,^ 12. Nesse cenário, a desvalorização dos periódicos brasileiros se manifesta através da baixa quantidade de citações envolvendo artigos publicados em revistas brasileiras em comparação a textos de revistas internacionais, com justificativa subentendida de que há superioridade qualitativa de artigos estrangeiros^13^.

Portanto, após a realização de revisão da literatura, não foram encontrados trabalhos recentes que comprovem e quantifiquem o desbalanço entre a citação de artigos internacionais e nacionais nos periódicos brasileiros favorecendo periódicos de outras nações. Dessa forma, o objetivo deste estudo foi verificar a diferença entre o número de citações de periódicos nacionais e internacionais em três revistas brasileiras no período de 2016 a 2020.

## METODOLOGIA

Para a realização desse estudo, caracterizado como transversal e observacional, foram analisadas as referências de três periódicos brasileiros de cirurgia: Revista do Colégio Brasileiro de Cirurgiões (CBC), Jornal Vascular Brasileiro (JVB) e Acta Cirúrgica Brasileira (ACB).

Foram analisados todos os artigos publicados nessas revistas no intervalo de 2016 a 2020. Em relação aos critérios de inclusão, foram consideradas as referências de todos os artigos originais. Como critérios de exclusão, os estudos classificados como editoriais, relatos de caso, notas técnicas, desafios terapêuticos, erratas e revisões de literatura foram excluídos da análise.

Posteriormente, os estudos selecionados tiveram suas referências avaliadas, sendo contabilizado o número total de citações e se essas eram provenientes de periódicos brasileiros ou de periódicos estrangeiros, assim como a relação proporcional entre elas. Foram, ainda, desconsideradas referências provenientes de livros, páginas da internet, dissertações e citações de citações (apud).

No que diz respeito à análise estatística, foi utilizado o teste de Kruskal-Wallis para verificação da diferença entre os grupos, sendo considerado significativo o valor de p < 0,05. Por serem revistas de domínio público, não foi necessário submeter o projeto ao Comitê de Ética em Pesquisa.

## RESULTADOS

Após a coleta de dados, foram analisados 902 artigos dos três periódicos selecionados, totalizando 23.394 referências, com média de 25,81 ± 8,59 por artigo. Nesse contexto, 2.680 (11,45%) dessas eram nacionais, correspondente a uma média de 2,95 ± 3,79 referências brasileiras por artigo.

Em relação aos anos selecionados, 2016 foi o ano com maior número total de estudos (204) e citações (5.087). Logo após, o ano de 2018 apresentou 196 artigos e 4.995 referências, seguido de 2017, com 180 artigos e 4.610 referências; de 2019, com 174 estudos e 4.477 citações; e de 2020, com 148 pesquisas e 4.225 citações. No que diz respeito à proporção entre citações nacionais e estrangeiras, o ano de 2016 apresentou maior predominância das brasileiras, correspondendo a 12,67% do total, enquanto o ano de 2020 apresentou o menor índice (10,88%).

No que tange aos periódicos, conforme demonstrado nas Tabelas 1 e 2 e Figura 1, a ACB apresentou o maior número de artigos e citações no período analisado, correspondente a 507 (56,20%) artigos e 13.835 (59,13%) das referências avaliados no protocolo de pesquisa. Além disso, o periódico obteve a menor proporção de uso de pesquisas brasileiras (8,40%) quando comparado às demais revistas, apresentando 438 (86,39%) dos artigos com 0 a 20% de referências de revistas nacionais, com significância estatística (p < 0,05).

**Tabela 1:** Quantidade de artigos, referências totais e referências nacionais dos periódicos avaliados (2016-2020)

**Ano**	**Referências nacionais**						**Total de referências**			**Total de artigos**		
	**ACB**		**JVB**		**CBC**		**ACB**	**JVB**	**CBC**	**ACB**	**JVB**	**CBC**
	**N**	**%**	**N**	**%**	**N**	**%**	**N**	**N**	**N**	**N**	**N**	**N**
2016	333	9,77	108	23,2	204	16,76	3.405	465	1.217	130	22	52
2017	226	9,10	77	17,03	243	14,49	2.481	452	1.677	93	21	66
2018	219	7,38	56	16,32	250	14,81	2.965	343	1.687	111	17	68
2019	171	6,38	111	23,17	222	16,84	2.680	479	1.318	98	19	57
2020	214	9,28	93	15,81	153	11,47	2.304	588	1.333	75	24	49
Total	1.163	8,40	445	19,12	1.072	14,82	13.835	2.327	7.232	507	103	292

Fonte: protocolo de pesquisa.

ACB = Acta Cirúrgica Brasileira; JVB = Jornal Vascular Brasileiro; CBC = Revista do Colégio Brasileiro de Cirurgiões; N = número.

**Tabela 2:** Referências nacionais em relação ao total dos periódicos avaliados (2016-2020).

**Referências nacionais em relação ao total (%)**	**ACB**		**JBV**		**CBC**	
	**N**	**%**	**N**	**%**	**N**	**%**
0,00	200	39,45	24	23,30	54	18,94
1,00-20,00	238	46,94	40	38,83	156	53,42
21,00-40,00	53	10,45	25	24,27	63	21,58
41,00-60,00	11	2,17	9	8,74	15	5,14
61,00-80,00	1	0,20	3	2,91	3	1,03
81,00-100,00	4	0,79	2	1,94	1	0,34
**Total**	**507**	**100,00**	**103**	**100,00**	**292**	**100,00**

Fonte: protocolo de pesquisa.

ACB = Acta Cirúrgica Brasileira; JVB = Jornal Vascular Brasileiro; CBC = Revista do Colégio Brasileiro de Cirurgiões; N = número.

**Figura 1:** Total de artigos e referências da Acta Cirúrgica Brasileira (2016-2020). Fonte: protocolo de pesquisa.

O JVB foi responsável por 103 (11,41%) artigos e 2.327 (9,94%) das referências totais da pesquisa, conforme demonstrado na Figura 2. Ademais, a revista apresentou a maior utilização de estudos nacionais (19,12%) em relação às demais, com 64 (62,13%) artigos apresentando 0 a 20% de suas referências advindas de periódicos nacionais.

**Figura 2:** Total de artigos e referências do Jornal Vascular Brasileiro (2016-2020). Fonte: protocolo de pesquisa.

Na Revista do CBC, foram coletados 292 (32,37%) estudos e 7.232 (30,91%) citações (Figura 3). No que diz respeito à proporção de referências brasileiras, esse periódico apresentou 11,47% de utilização, exibindo 210 (71,92%) artigos com 0 a 20% de suas referências de periódicos nacionais.

**Figura 3:** Total de artigos e referências da Revista do Colégio Brasileiro de Cirurgiões (2016-2020). Fonte: protocolo de pesquisa.

Vale a pena ressaltar, ainda, que 30,59% dos artigos analisados não possuíam nenhuma referência brasileira. Sobre os periódicos, 23,30% dos artigos do JVB e 17,80% dos artigos da Revista do CBC não citavam estudos nacionais. Acerca da Acta Cirúrgica Brasileira, a revista demonstrou a maior quantidade de artigos com referências totalmente estrangeiras (39,44%).

## DISCUSSÃO

O processo de produção científica brasileira é recente e ainda está em consolidação. Além da estruturação tardia do ensino universitário, a tradição pedagógica nacional ainda é deficitária e defasada ao privilegiar técnicas de exposição e memorização em detrimento de uma formação construtivista e crítica^14^. Não obstante, indicadores internacionais demonstram um avanço considerável do Brasil no campo das ciências ao longo das últimas quatro décadas: no começo dos anos 2000, entre os 30 países com maior destaque no *ranking* da ciência mundial no Institute for Scientific Information (ISI), obteve o sexto melhor desempenho, ficando atrás de Coreia do Sul, Taiwan, China, Espanha e Turquia^15^.

Na área médica, no entanto, as publicações científicas brasileiras tendem a conceder maior destaque para publicações internacionais, sobretudo em língua inglesa e de origem estadunidense, em detrimento da produção nacional. Mesmo com publicações relevantes, os periódicos do Brasil são pouco lidos, mantendo baixo FI no cenário internacional^16^^,^ 17.

O presente estudo corrobora a manutenção dessa tendência nos últimos anos. De fato, entre os periódicos analisados, houve uma queda nas citações nacionais, passando de 12,67% em 2016 para 10,88% em 2020. Mesmo no periódico com maior proporção de referências brasileiras (Jornal Vascular Brasileiro), publicações internacionais representaram ampla maioria (80,88%).

Sobre esse tema, Petroianu^18^ destaca haver um grande número de trabalhos de alta qualidade em periódicos nacionais que não conseguem atenção sequer dos brasileiros, o que classifica como uma tendência colonialista e autofágica. Aprofundado sua análise, acrescenta que os autores brasileiros, mesmo patrocinados com verbas nacionais, acabam por beneficiar outros países ao privilegiar seus periódicos, sem nenhuma contrapartida para a ciência local. Deve-se considerar que esse padrão é reforçado pelos próprios órgãos encarregados de avaliar instituições científicas brasileiras, ao priorizarem o FI das revistas em que seus estudos são publicados e desencorajarem, por conseguinte, publicações em periódicos nacionais menores.

A respeito das consequências desse desprestígio, Meneghini^19^ argumenta que, embora o inglês seja o idioma dominante, sua utilização pode dificultar o acesso de publicações na área biomédica por parte de leitores e usuários, como profissionais de saúde e jornalistas, em especial nos países em desenvolvimento. A dificuldade no entendimento de publicações internacionais é particularmente preocupante em estudos com implicações clínicas, já que pode privar os pacientes desses profissionais de eventuais benefícios. Ademais, deve-se considerar a discrepância das produções científicas nacional e internacional, principalmente no que se refere a áreas específicas da literatura, uma vez que essa desproporção privilegia a citação de artigos internacionais, devido à vasta produção quando comparada à nacional.

Outro problema diz respeito à tendência de se ignorar publicações de alta relevância em função do idioma. A título de exemplo, cientistas alemães já haviam identificado uma relação causal significativa entre o tabagismo e o câncer de pulmão no início dos anos 1930, achado ignorado pela comunidade científica por mais de três décadas, até que cientistas britânicos e estadunidenses redescobrissem essa ligação e fomentassem políticas públicas contra o cigarro^20^.

Em estudo similar ao presente, Teixeira^21^ pontua que, embora a prática de citar periódicos internacionais não possa ser considerada como errada, trata-se de um ciclo perpétuo de desprestígio para os pesquisadores nacionais. Acrescenta, ainda, que o FI dos periódicos nacionais analisados na ocasião cresceu entre os anos de 2007 e 2011, o que reforça o elevado grau de qualidade da pesquisa brasileira. Além disso, a falta ou a indexação recente de revistas nacionais em bases de busca de como a PubMed, desenvolvida pelo National Center for Biotechnology Information (NCBI), e outras agrava o problema de visibilidade da produção científica nacional, impedindo o alcance dessa literatura tanto aos pesquisadores nacionais quanto internacionais, diminuindo o impacto da produção brasileira.

A existência de uma “língua franca” não é novidade ao longo da história da humanidade, sobretudo por razões comerciais e diplomáticas. Ainda hoje, o francês, idioma da diplomacia na Europa do século XVII, é utilizado em instituições internacionais. Assim, ao facilitar a comunicação entre pesquisadores, o aprendizado da língua inglesa deve ser encorajado na comunidade científica^22^.

Por outro lado, encorajar o entendimento de periódicos internacionais não significa ignorar a necessidade de prestígio à linguagem científica nativa. Nesse sentido, a publicação de artigos bilíngues e multilíngues é uma solução em potencial, pouco custosa e oferecida há anos pela Scientific Electronic Library On-line (SciELO), biblioteca eletrônica fundada em 1997 pela Fundação de Amparo à Pesquisa do Estado de São Paulo (FAPESP) em parceira com o Centro Latino-Americano e do Caribe de Informação em Ciências da Saúde (BIREME)^23^.

Idealmente, tanto periódicos nacionais quanto internacionais deveriam considerar a publicação de ao menos duas versões dos artigos – uma em inglês e outra na língua nativa do autor. São necessários esforços conjuntos por parte de agências nacionais, editores e autores para ampliar as publicações bilíngues e multilíngues e aumentar a visibilidade da pesquisa brasileira. Não obstante, os próprios autores brasileiros podem e devem empreender esforços para referir trabalhos nacionais, quando forem pertinentes, cabendo ao corpo editorial das revistas brasileiras atentar para corrigir omissões no que tange às citações de estudos locais.

## CONCLUSÃO

É evidente o crescimento qualitativo que a pesquisa brasileira teve nos últimos anos; entretanto, ainda há diferença significativa entre o número de citações de periódicos internacionais em detrimento dos nacionais. Nessa perspectiva, se faz necessária maior valorização dos periódicos brasileiros, em especial por parte dos pesquisadores nacionais e das instituições responsáveis pelo fomento da ciência, a fim de desenvolver, cada vez mais, o incentivo à produção do conhecimento.
